# Genetic Mapping by 55K Single-Nucleotide Polymorphism Array Reveals Candidate Genes for Tillering Trait in Wheat Mutant *dmc*

**DOI:** 10.3390/genes15121652

**Published:** 2024-12-22

**Authors:** Kemeng Jiao, Guojun Xia, Yuan Zhou, Chenyu Zhao, Huiyuan Yan, Menglei Qi, Pingfan Xie, Yongjing Ni, Jingxue Zhao, Jishan Niu, Zhaofei Chao, Jiangping Ren, Lei Li

**Affiliations:** 1 Henan Technology Innovation Centre of Wheat/National Engineering Research Centre for Wheat, Henan Agricultural University, Zhengzhou 450046, China; j548654@163.com (K.J.); gmgs002@163.com (G.X.); zy200102242023@163.com (Y.Z.); 13782885547@163.com (C.Z.); 13523408147@163.com (H.Y.); 15639491503@163.com (M.Q.); 18530091591@163.com (P.X.); 18625453972@163.com (J.Z.); jsniu@henau.edu.cn (J.N.); xmzxrjp@126.com (J.R.); 2 National Key Laboratory of Wheat and Maize Crop Science, College of Agronomy, Henan Agricultural University, Zhengzhou 450046, China; 3 Henan Engineering Research Centre of Wheat Spring Freeze Injury Identification, Shangqiu Academy of Agriculture and Forestry Sciences, Shangqiu 476000, China; nyj317@163.com; 4 Jiaozuo Seed Industry Development Center, Jiaozuo 454150, China; 13903895296@139.com

**Keywords:** *dmc*, tillering mutant, 55K SNP array, gene mapping

## Abstract

Background: The tiller number is a key agronomic trait for increasing the yield potential of wheat (*Triticum aestivum* L.). A number of quantitative trait loci (QTLs) and key genes controlling tillering have been identified, but the regulatory mechanisms remain unclear. Methods: In this study, we utilized the dwarf-monoculm mutant (*dmc*) obtained from the ethyl methane sulfonate (EMS)-treated wheat cultivar Guomai 301. The F_2_ populations were constructed using the *dmc* mutant crossed to multiple tiller parents. The F_2_ populations were surveyed for tillering traits at the critical fertility stage for genetic analyses. The extreme-tillering-phenotype plants from the F_2_ population were used to construct mixing pools that were analyzed by a wheat 55K SNP array. The tillering genes of *dmc* were mapped using the wheat 55K SNP array combined with transcriptomic data. Results: The results showed that the genetic phenotype of *dmc* is controlled by two dominant genes. The tillering genes of *dmc* were mapped on the 60–100 Mb region of chromosome 5B and the 135–160 Mb region of chromosome 7A. A total of sixteen candidate genes associated with the tillering trait of *dmc* were identified. Two candidate genes, TraesCS5B02G058800 and TraesCS7A02G184200, were predicted to be involved in indole acetic acid (IAA) response and transport, which were considered as potential regulatory genes. Conclusions: This study elucidated the genetic basis of the *dmc* mutant and provided two valuable reference genes for studying the development and regulatory mechanisms of wheat tillering.

## 1. Introduction

Wheat plays a crucial role in global food security, serving as a vital food crop that feeds billions of people worldwide [1,2,3]. According to relevant studies, global grain trade will be affected by both climate extremes and population growth, with a reduction in wheat supply capacity in key supply regions [4,5,6,7]. Tillering is a critical aspect of plant development, particularly in grasses and cereal crops, as it significantly influences biomass production and yield [8]. Tiller formation has a significant effect on wheat in terms of building a rational population, increasing the number of effective spikes and grain yield [9,10]. Therefore, it is necessary to excavate the key genes of tiller regulation and study the mechanism of tiller formation in wheat.

The regulation of tillering involves a complex interplay of genetic factors, hormonal signaling, and environmental conditions [11,12,13]. The continued growth of tillers requires a steady supply of sugar. The early cessation of tillering in the *tiller inhibition mutant (tin)* mutant leads to a reduction in the number of tillers [14]. High-density planting reduces photosynthetically active radiation (PAR) intensity and causes the earlier cessation of tiller development [8]. Cytokinin (CK)-mediated signaling effects of fertilizer nitrogen forms can be employed as a management tool to regulate the tiller number in cereal crops [15]. Gibberellins (GAs) and strigolactones (SLs) are the two major phytohormones determining plant tillering, *Oryza sativa GROWTH-REGULATING FACTOR7(OsGRF7)* alters the endogenous strigolactone content, which rendered repression of the outgrowth of the axillary buds [16]. The *TILLER NUMBER 1(TN1)* gene promotes wheat tillering, at least partially, through two layers of molecular mechanisms via repressing abscisic acid (ABA) biosynthesis and inhibiting ABA signaling through preventing the binding of *PYR-like (TaPYL)* to *PROTEIN PHOSPHATASE 2C (TaPP2C)* [17]. Gibberellins also play a role in stimulating tiller development, although their effects can vary between species [18,19]. Previous correlative studies of *dmc* mutants have shown that tillering is associated with the IAA biosynthesis pathway [20]. Studying the *dmc* mutant could enhance our understanding of the complex regulatory network of tillering development.

Tillering originates from the axillary meristem (AM), and its formation and development primarily occur in two stages: (1) the development of the axillary meristem, which begins with the formation of a boundary between the shoot apex and the leaf primordia, followed by the initiation of meristematic cells in the axillary region near the leaf axil; (2) the elongation of the axillary bud, leading to the formation of tillers [21,22]. Many crops with tiller-related genes have been reported. Regulator genes in rice (*Oryza sativa* L.) influencing AM formations include *O. sativa homeobox1* (*OSH1*) [23], *LAX PANICLE1* (*LAX1*) [24], LAX PANICLE*2 (LAX2)* [25], *MONOCULM1* (*MOC1)* [26,27,28], *MOC1 interacting protein1 (MIP1)* [29], *Tillering and Dwarf1 (TAD1)* [30], *SLENDER RICE1 (SLR1)* [28], *MONOCULM3 (MOC3)* [31], *RICE FLORICULA/LEAFY (RFL)* [32], *Oryza sativa CUP-SHAPED COTYLEDON1 (OsCUC1)* [33], and *FRIZZLE PANICLE* (*FZP)* [34], while regulator genes influencing AM growth into tillers include *DWARF3* (*D3)* [35], *DWARF10 (D10)* [36], *DWARF14 (D14)* [37], *HIGH TILLERING DWARF1 (HTD1)* [38], *DWARF27 (D27)* [39], *O. sativa MORE AXILLARY GROWTH1a (OsMAX1a)* [40], *O. sativa MORE AXILLARY GROWTH1e (OsMAX1e*) [40], *DWARF53 (D53)* [37], *(O. sativa MINICHROMOSOME MAINTENANCE1*, *AGAMOUS*, *DEFICIENS and SERUM RESPONSE FACTOR57) (OsMADS57)* [41], *O. sativa TEOSINTE BRANCHED1 (OsTB1)* [41], *IDEAL PLANT ARCHITECTUTRE1 (IPA1)* [42], *O. sativa DENSE AND ERECT PANICLE1 (DEP1)* [42], *O. sativa SHORT INTERNODES1OsDEP1 (OsSHI1)* [42], *O. sativa CIRCADIAN CLOCK ASSOCIATED1 (OsCCA1)* [43], *Heading date 3a (Hd3a)* [44], *O. sativa Domains Rearranged Methyltransferase2* (*OsDRM2)* [45], *FLORAL ORGAN NUMBER1 (FON1*) [31], *TILLER NUMBER 1 (TN1)* [46], and *TN1 interaction factor 1 (TIF1)* [46]. Regulator genes in wheat regulating tillering include *TILLER NUMBER 1 (TN1)*, *tiller inhibition mutant (tin)*, *tin2*, *tin3*, *tin4*, *tin5*, *tin6*, *fertile tiller inhibition gene (ftin)*, and *oligo-tillering mutant (ot1)*, having diverse effects on axillary bud development and overall wheat growth [8,14,47,48,49,50,51,52].

Tillering mutants are crucial for understanding the genetic and physiological mechanisms that regulate tillering in various plant species, particularly in crops like wheat and rice. These mutants provide valuable insights into the factors that influence tiller development. Morphological studies indicate that mutants like *tin*, *tin4*, *tin5*, *ftin*, *and TN1* exhibit decreased tiller numbers due to inhibited or abnormal axillary bud development [17,47,48,49,53]. In *tin* mutants, tiller growth ceases when the plant transitions from the vegetative to the reproductive stage [14,54,55]. The *ftin* mutants show normal tiller numbers at the seedling stage, but exhibit a significant reduction at heading, suggesting that the reduction is due to delayed tiller outgrowth rather than inhibited axillary bud differentiation [49]. In *tin4*, *tin5*, and *TN1*, the decreased tiller number results from the inhibition of secondary tiller bud development [17,47,48]. The ot1 mutant maintained an average of three tillers [52]. Meanwhile, in *dmc*, a non-tillering mutant from EMS-treated wheat cultivar Guomai 301, both tiller bud differentiation and development are inhibited [56,57]. This means that our material, *dmc* mutants, is different from the mutants that have been reported.

In this study, we combined transcriptomic information by wheat 55K SNP array sequencing [56], and sixteen candidate genes were predicted within the two target interval segments. Two of them, TraesCS5B02G058800 and TraesCS7A02G184200, are tillering regulatory genes, which were predicted to be involved in IAA response and transport.

## 2. Materials and Methods

### 2.1. Plant Material

The dwarf-monoculm mutant *dmc* was obtained from an EMS-mutagenized population of the wheat variety Guomai 301. Through consecutive years of single-plant selection, we successfully obtained genetically stable strains of *dmc*. The hybridization of the *dmc* mutant with Aikang 58, Chinese Spring, Guomai 301, Zhengmai 379, and Zhengmai 9405 produced 15, 6, 17, 27, and 21 F_1_ individuals, respectively. The F_1_ individual, self-pollinated *dmc* mutant and Aikang 58, Chinese Spring, and Guomai 301 produced 460, 811, and 1041 F_2_ individuals, respectively. These F_1_ individuals and F_2_ populations were planted in Xiaowu Village, Yuanyang City, Henan, China (35°6′ N, 113°56′ E, 70 m a.s.l.). All materials were sown in mid-October and harvested in June of the following year in 2023 and 2024 wheat growth seasons. All F_1_ and F_2_ individuals were sown in 2 m rows with 25 cm row spacing, leaving approximately 10 cm between plants. Varieties were obtained from the National Engineering Research Centre for Wheat. Fertilizer and weed management were similar to wheat breeding [58].

### 2.2. Phenotypic Investigations and Genetic Analysis

At least 30 plants of the *dmc* mutant and wheat cultivar Guomai 301 were surveyed phenotypically at the tillering stage and filling stages. A statistical analysis was conducted using Origin 2022 software. All lines of the F_2_ population were surveyed for the tiller number in each April of 2023 and 2024. The data were statistically analyzed using SPSS v20 software.

### 2.3. Cytological Karyotype Analysis

Chromosome configurations and a high-resolution chromosome painting analysis of plants derived from the *dmc* mutant at the metaphase of mitosis: Chromosome samples were prepared as previously described [59]. For chromosome painting, eight single-strand oligonucleotides were used to form modified multiplex probes for the karyotype analysis in wheat, which included TAMRA (6-carboxytetramethylrhodamine)-modified oligonucleotides pAs1-1, pAs1-3, pAs1-4, pAs1-6, AFA-3, and AFA-4, and two FAM (6-carboxyfluorescein)-modified oligonucleotides, pSc119.2-1 and (GAA)10 [59]. Oligonucleotide probes used for FISH are listed in Table S1. The FISH procedure was tested as previously described [59]. Chromosomes were visualized with microscope Olympus BX51 (Olympus, Beijing, China) and pictures were captured with SPOT CCD (SPOT Cooled Color Digital Camera, Leica, Germany). An image analysis was conducted using Photoshop v6.0.

### 2.4. Wheat 55K SNP Array Analysis

Nine no-tillering and thirty multi-tillering individuals with extreme phenotypes from the Aikang58 × *dmc* F_2_ populations were prepared to construct two pools. Genomic DNA was extracted by Nakayukin Marker Company (http://www.cgmb.com.cn/, accessed on 26 April 2023) (Beijing, China). The bulked samples were genotyped with the Axiom^®^ Wheat 55K SNP array. The work was mainly conducted at Nakayukin Marker Company (http://www.cgmb.com.cn/, accessed on 26 April 2023). We performed an SNP gene analysis and cluster analysis using Biosearch’s high-throughput genotyping assay platform (https://www.biosearchtech.com/, accessed on 26 April 2023). We excluded SNP markers with deletion rates greater than 20%, allele frequencies less than 5%, and those that could not be localized to chromosomes. We screened SNP markers with differences in the mixed pools that corresponded to the extreme phenotypes. We compared and analyzed the sequences of the selected SNP markers with the Chinese Spring Genome Version 1.0 (http://www.wheatgenome.org/, accessed on 26 April 2023) for chromosome localization.

### 2.5. Expression Analysis

We collected tiller nodes from the *dmc* and Guomai 301 varieties separately. RNA was extracted using a Trizol reagent (TransGen Biotech, Beijing, China) according to the manufacturer’s protocol. RNA concentration was measured using a NanoDrop 2000 (NanoDrop Technologies, Wilmington, DE, USA). RNA integrity was assessed using the RNA Nano 6000 Assay Kit on the Agilent Bioanalyzer 2100 system (Agilent Technologies, Santa Clara, CA, USA). We used Primer5 software to design gene-specific primers (Table S2). The wheat actin gene was used as an internal control gene. The qRT-PCR reactions were performed in 20 µL volumes containing 10 µL Hieff^®^ qPCR SYBR Green Master Mix (Yeasen Biotech, Shanghai, China), 0.8 µL primer mix (10 µM), 1 µL cDNA (50 ng), and 8.8 µL ddH_2_O. The PCR parameters were 94 °C for 30 s, and then 40 cycles of 94 °C for 5 s, and 60 °C for 30 s. All qRT-PCR reactions were replicated three times. The gene expression levels were calculated according to the 2^−∆∆Ct^ method [60].

## 3. Results

### 3.1. Identification of dmc Mutants

**Figure 1:**
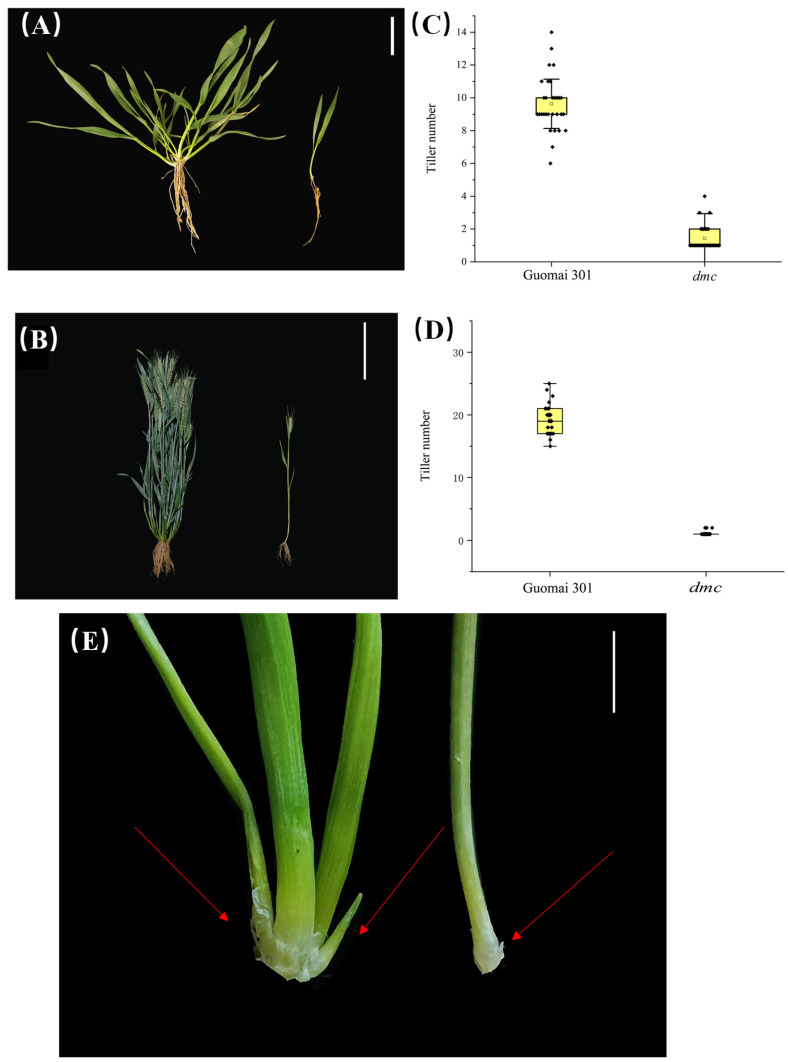
Phenotypes of the *dmc* mutant; (**A**) Guomai 301 (left) and the *dmc* mutant (right) at the tillering stage, and the scale bar is 5 cm; (**B**) Guomai 301 (left) and the *dmc* mutant (right) at the filling stage, and the scale bar is 10 cm; (**C**) the number of tillers at the tillering stage of Guomai 301 and the *dmc* mutant, and the data are the mean ± SD (*n* = 30 biologically independent samples); (**D**) the number of tillers at the filling stage of Guomai 301 and the *dmc* mutant, and the data are the mean ± SD (*n* = 30 biologically independent samples); (**E**) the tiller buds at the three-leaf stage of Guomai 301 (left) and the *dmc* mutant (right), the scale bar is 0.5 cm, and red arrows indicate the position of axillary buds.

The *dmc* mutant was previously characterized from the EMS-treated wheat cultivar Guomai 301. At the tillering stage, Guomai 301 exhibited 9–12 tillers, whereas the *dmc* mutant displayed only one main stem (Figure 1A,C). At the filling stage, Guomai 301 produced approximately 20 tillers, while the *dmc* mutant developed only one main stem (Figure 1B,D). During the three-leaf stage, the *dmc* mutant presented a small tiller bud compared to the multiple tiller buds of Guomai 301 (Figure 1E).

### 3.2. Fluorescence In Situ Hybridization Analysis of dmc Mutant Chromosomes

**Figure 2:**
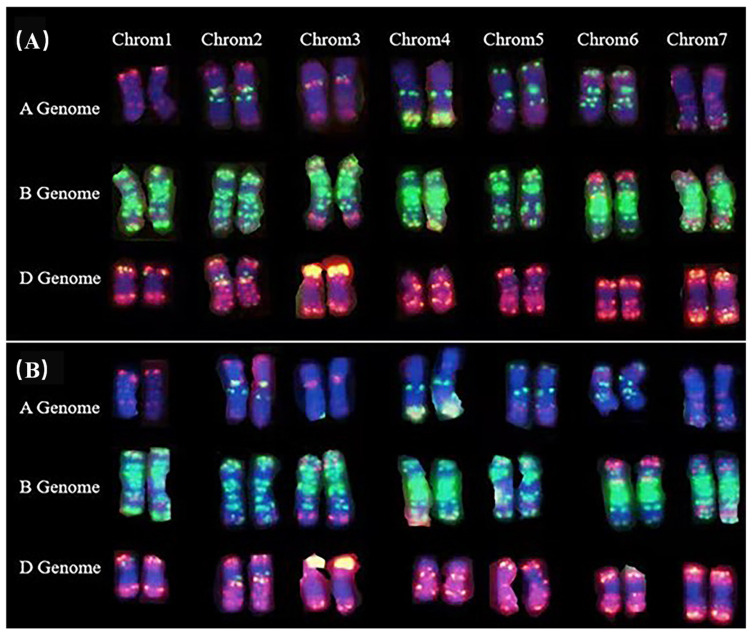
The high-resolution chromosome painting analysis of the plants derived from the *dmc* mutant at the metaphase of mitosis. (**A**) One karyotype of Guomai 301, which had 42 normal chromosomes. (**B**) One karyotype of the *dmc* mutant, which had 42 normal chromosomes. Blue color, chromosomes counterstained with DAPI; green, signals of oligos pSc119.2-1, (GAA)10; red, signals of oligos AFA-3, AFA-4, pAs1-1, pAs1-3, pAs1-4, and pAs1-6. Scale bar = 10 µm.

To validate and identify the chromosome constitution of the *dmc* mutant, high-resolution chromosome painting was applied using eight single-strand oligonucleotide probes. The result of the fluorescence in situ hybridization analysis of chromosomes revealed that the A, B, and D genomes of the *dmc* mutant were structurally normal. There is no large segment deletions compared to the control Guomai 301 (Figure 2A,B). This finding indicates that the phenotype of the *dmc* mutant is not attributable to chromosomal structural variations.

### 3.3. Genetic Analysis of Tiller Number of dmc Mutant

The *dmc* mutant was crossed with several multi-tiller parents (Table 1). The F_1_ generation was examined for a genetic analysis of the tiller number associated with the *dmc* mutant. The tiller count in the F_1_ generation was intermediate between that of the *dmc* mutant and the multi-tiller parents. This suggests that the *dmc* mutant may be regulated by a semi-dominant gene.

The *dmc* mutant showed only one main stem but with an ineffective tiller. In the experiment, the number of effective tillers greater than one was defined as multiple tillers, and only one main stem was defined as a no-tiller plant. Assuming that the no-tiller trait is controlled by two genes, the survey showed that the F_2_ populations of *dmc* × Aikang 58 in 2023, *dmc* × Chinese Spring in 2023 and 2024, and *dmc* × Guomai 301 in 2024 had 36, 27, 20, 21, and 50 no tillers, respectively (Table 2). The multiple tillers had 424, 396, 368, 380, and 385 tillers, respectively (Table 2). The result of the χ^2^ test showed that the number of effective tillers was χ^2^ < *P*_0.05_, which is consistent with the Mendelian two-gene inheritance of a 15:1 segregation ratio. It was concluded that the no-tiller phenotype of the *dmc* mutant was controlled by two genes.

**Figure 3:**
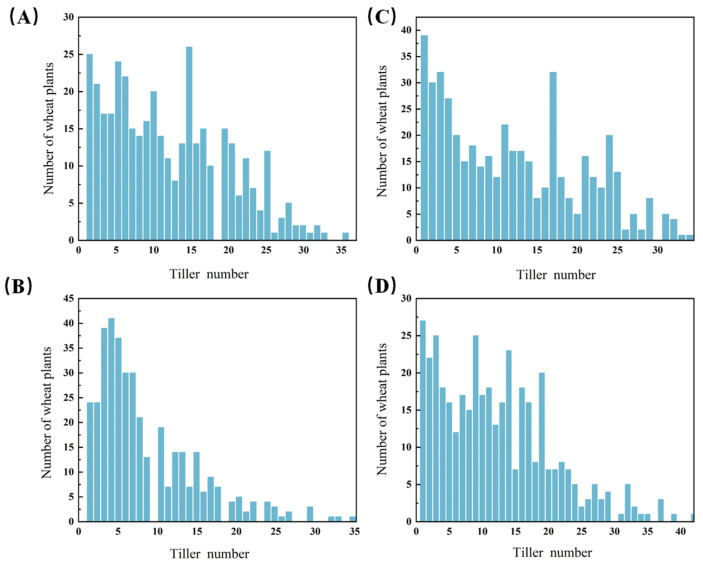
The effective tiller number and distribution frequency of the F_2_ population; (**A**) the F_2_ population of *dmc* × Guomai 301 in 2023; (**B**) the F_2_ population of *dmc* × Aikang 58 in 2023; (**C**) the F_2_ population of *dmc* × Chinese Spring in 2024; (**D**) the F_2_ population of *dmc* × Chinese Spring in 2023.

It shows that in the F_2_ populations from the crosses of *dmc* × Chinese Spring, *dmc* × Aikang 58, and *dmc* × Guomai 301, a greater number of plants exhibited fewer tillers than those with a higher number of tillers (Figure 3). This observation supports the involvement of semi-dominant genes in the F_1_ generation. We supposed that two semi-dominant genes regulate the no-tillering trait. The number of dominant genes associated with this trait varies by phenotype. For instance, the AABB genotype is characterized by the no-tillering trait.

### 3.4. Analysis of Gene Microarrays

**Figure 4:**
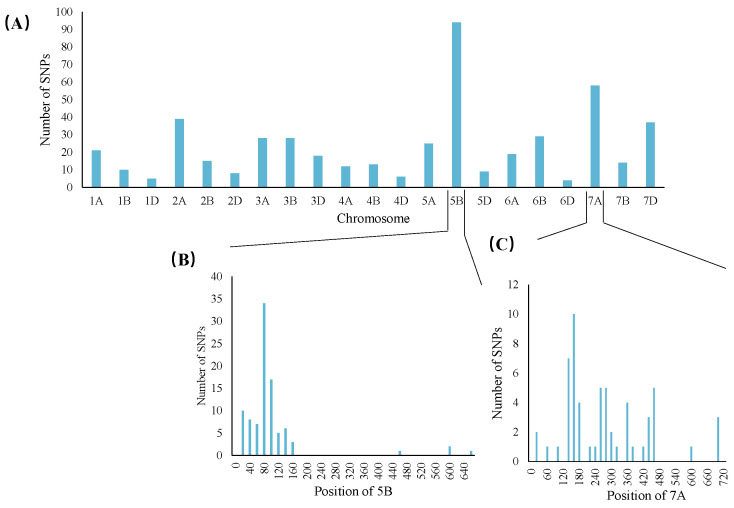
Distribution of single-nucleotide polymorphisms (SNPs) on different chromosomes and polymorphic SNP sites on chromosomes 5B and 7A in wheat; (**A**) number of polymorphic SNPs on each chromosome; (**B**) distribution of polymorphic SNP sites on chromosome 5B; (**C**) distribution of polymorphic SNP sites on chromosome 7A.

An F_2_ population consisting of 460 individuals derived from the cross between Aikang58 and the *dmc* mutant was used for the analysis. We first conducted a bulked segregant analysis and wheat 55K SNP array-based genotyping using the individuals with extreme phenotypes from the F_2_ progenies. A total of 491 SNPs were polymorphic, of which 169 were located on chromosome 5B and 58 were located on chromosome 7A (Figure 4A). A further analysis showed that 58 SNPs are enriched over a 60 to 100 Mb interval on the short arm of chromosome 5B, and 21 SNPs are enriched over a 135 to 160 Mb interval on the short arm of chromosome 7A (Figure 4B,C).

### 3.5. Candidate Gene Prediction

Combined with the transcriptome data, the differential genes contained in the two candidate intervals were analyzed and annotated [56,61]. A total of sixteen differentially expressed genes were identified (Table 3). According to transcriptome data, seven candidate genes on chromosome 5B and TraesCS7A02G184200 are upregulated in the *dmc* mutant, while the remaining eight genes on chromosome 7A are downregulated in the *dmc* mutant. The gene annotation revealed that TraesCS5B02G058800 is involved in regulating growth hormone response proteins. And TraesCS7A02G184200 encodes a protein featuring the BTB structural domain, which plays a role in the transport of growth hormones. This finding aligns with our previous studies where IAA metabolism and signaling affected tillering development of the *dmc* mutant [20]. Consequently, TraesCS5B02G058800 and TraesCS7A02G184200 are two potential candidate genes regulating tillering development for further research.

**Figure 5:**
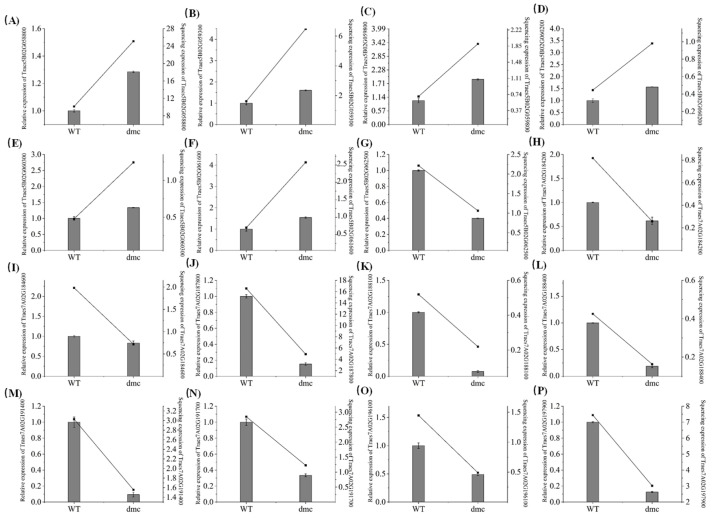
The qRT-PCR analysis of sixteen candidate genes. (**A**) TraesCS5B02G058800 (Auxin-responsive protein IAA31); (**B**) TraesCS5B02G059300 (CBL-interacting protein kinase 4); (**C**) TraesCS5B02G059800 (Tuliposide A-converting enzyme 2); (**D**) TraesCS5B02G060200 (Dolabradiene monooxygenase); (**E**) TraesCS5B02G060300 (Dolabradiene monooxygenase); (**F**) TraesCS5B02G061600 (11-beta-hydroxysteroid dehydrogenase A); (**G**) TraesCS5B02G062500 (Methylcrotonoyl-CoA carboxylase subunit alpha); (**H**) TraesCS7A02G184200 (Protein containing a BTB complex and an NPH3 domain (BTBN), regulation of auxin transport); (**I**) TraesCS7A02G184600 (Shikimate kinase domain-containing protein); (**J**) TraesCS7A02G187800 (Aquaporin NIP III subfamily protein); (**K**) TraesCS7A02G188100 (Amino acid transporter); (**L**) TraesCS7A02G188400 (Plant regulator RWP-RK domain-containing protein); (**M**) TraesCS7A02G191400 (Small GTP-binding protein OsRac3); (**N**) TraesCS7A02G191700 (Domain of unknown function DUF23); (**O**) TraesCS7A02G196100 (TGF-beta receptor, type I/II extracellular region family protein); (**P**) TraesCS7A02G197900 (Similar to CEL5 = CELLULASE 5). The left y-axis represents relative expression, and relative expressions are shown by histograms. The right y-axis represents the expression value (FPKM) of transcriptome sequencing, and expression values are shown by lines. Error bars indicate the standard deviation. The x-axis indicates the samples of Guomai 301 and *dmc*.

We performed a qRT-PCR analysis of sixteen candidate genes’ expression during the tillering stage of Guomai 301 and *dmc* for the validation of transcriptome data (Figure 5). It showed that the qRT-PCR results matched well with the transcriptome data.

## 4. Discussion

Increasing wheat yield is an important means to cope with future food shortages [62,63,64]. It is an effective way to increase wheat yield to study the mechanism of tillering control and increase the amount of effective tillering [47,65]. In the *dmc* mutant, the growth of tiller buds and plant development are inhibited (Figure 1A,B). Most of the primordia could not grow or stopped developing, which resulted in no tillers [56].The *dmc* mutant exhibits a dwarf phenotype with no tillers, making it an excellent material for studying tiller suppression.

Previous studies have reported eight tillering inhibition genes of wheat (*tin1*, *tin2*, *tin3*, *ftin*, *TIN4*, *TIN5*, *tin6*, *TN1)* [8,14,17,47,48,49,50,51]. Among these, the natural mutants *tin, tin2*, *ftin*, and *TIN4* are located on 1AS, 2A, 1AS, and 2DL, respectively [8,14,47,49]. The EMS-treated mutants *tin3, TIN5, tin6*, and *TN1* are located on chromosomes 3A, Tu7, 2DL, and 6BS, respectively [8,17,48,50,51]. The QTL associated with the tiller number in wheat is located on chromosomes 1A, 1B, 2B, 2D, 4A, 4B, 4D, 5A, 6B, and 7D [66,67,68,69,70,71,72]. In this study, we predicted two new wheat tiller inhibition genes, whose physical locations are different from those of *pin-formed 1* (*TaPIN1)* [73], *dwarf (TaD27-B)* [39], *ovarian tumor domain-containing proteases 1 (TaOTUB1)* [74], and *squamosa promoter binding-like 14* (*TaSPL14)* [75] found previously on chromosomes 5B and 7A. This study contains a new finding of a tillering inhibition mutant (Figure 4B,C).

The downregulated gene expressions related to phytohormone syntheses of auxin, zeatin, cytokinin, and some transcription factor (TF) families of TALE, and WOX, might be the major causes of non-tillering in mutant *dmc* [20,56,57,76]. The gene *tin* reduces tillering by controlling the early maturation of internodes, causing a competition for sucrose between the internodes and tiller buds [14,53]. Similarly, *TN1* inhibits tillering by regulating the levels of ABA [17]. The *ot1* gene revealed the upregulation of genes associated with SL and ABA biosynthesis and signaling [52]. Previous studies have proved that IAA contents in *dmc* were significantly less than that in Guomai 301 at tillering stages [56]. In this study, TraesCS5B02G058800 and TraesCS7A02G184200 are related to IAA regulation and transport among the sixteen candidate genes identified in the positional cloning prediction. We predict that they are candidate genes of non-tillering in the *dmc* mutant (Table 3).

Using homologous gene cloning methods, several genes related to wheat tillering have been identified [77,78]. These genes act at different developmental stages of wheat growth and participate in the regulation of tillering through interactions with various genetic factors or exogenous hormones. For instance, *MOC1* encodes a putative GRAS family nuclear protein that is expressed mainly in the axillary buds and functions to initiate axillary buds and to promote wheat outgrowth [28]. In contrast, the *TaTB1* gene overexpression in wheat results in reduced tillers and spike numbers [79]. Additionally, *phytochrome-interacting factor-like 1* (*TaPIL1)* activates the transcriptional expression of wheat *TaTB1*, reducing the wheat tiller number [80]. Moreover, *the PLANT ARCHITECTURE AND YIELD 1 (TaPAY1)* gene could improve the tiller number via affecting polar IAA transport activity and altering endogenous IAA distribution [81]. The *TaPIN1s* indicating that IAA might mediate the axillary bud production and reduction in *TaPIN1* expression increased the tiller number and grain yield per plant of wheat [73,82,83]. The application of SL inhibits shoot branching in plants [84]. The *TaD27-B* modulates tillering by participating in the biosynthesis of SL, while *TaD53* acts as a repressor in the SL signaling pathway, interacting with the transcriptional co-repressor Topless (*TaTPL)* [39]. The *TERMINAL FLOWER 1 (TaTFL1)* was implicated in tiller regulation by IAA and CK signaling [85]. These examples illustrate the complexity of factors regulating wheat tillering. However, only the mechanisms of ABA and SL in tiller regulation have been reported, while the role of IAA in this process remains unclear. Our previous studies have shown that IAA expression in *dmc* is suppressed, which is a critical factor causing the dwarfing and no tillering in this mutant [20,56]. Therefore, *dmc* provides a valuable new mutant material for studying the mechanisms of tillering in wheat, holding significant implications for molecular breeding in the crop.

## 5. Conclusions

In this study, F_1_ individuals and F_2_ populations of the *dmc* mutant obtained by multi-tillering varieties were constructed for a genetic analysis and oligotillering gene mapping. The populations showed that the genetic phenotype of the *dmc* mutant was controlled by two dominant genes. The analysis of a wheat 55K SNP array showed that the *dmc* tiller gene was located on the 60–100 MB region of chromosome 5B and the 135–160 MB region of chromosome 7A. A total of sixteen candidate genes related to *dmc* tillering traits were identified in the candidate region by combining the wheat 55K SNP array results with the transcriptome information of *dmc* mutants. Among them, TraesCS5B02G058800 and TraesCS7A02G184200 genes are predicted to be involved in the response and transport of IAA, and are considered as potential regulatory genes of tillering. Cloning the *dmc* tillering gene could aid in developing tillering-related markers for wheat breeding and enhance our understanding of the biological mechanisms underlying this complex trait.

## Figures and Tables

**Table 1:** The amount of tillering in the F_1_ generation of the different hybrid combinations.

Hybrid Combination	Number of Individual F_1_ Plants	Number of Tillers of Multi-Tiller Parent	Number of Tillers in the F_1_ Generation ^1^
*dmc* × Zhengmai 9405	4	13 ± 1.48	9.75 ± 5.58
Zhengmai 9405 × *dmc*	17	13 ± 1.48	4.64 ± 1.93
*dmc* × Aikang 58	3	22 ± 2.23	5 ± 2
Aikang 58 × *dmc*	13	22 ± 2.24	5.53 ± 1.39
*dmc* × Zhengmai 379	17	14 ± 1.14	8.9 ± 3.73
Zhengmai 379 × *dmc*	10	14 ± 1.14	5.23 ± 2.07
*dmc* × Guomai 301	11	23 ± 2.22	11.75 ± 2.06
Guomai 301 × *dmc*	6	23 ± 2.22	6.18 ± 4.52

^1^ The data are the means ± SD.

**Table 2:** Genetic analysis of tillering traits in F_2_ populations.

Hybrid Combination	Year	Multiple Tillers	No Tiller	Expected Ratio	χ^2^	*p*-Value ^1^
					χ^2^ (15:1)	P *_0.05_
*dmc* × Aikang 58	2023	424	36	15:1	1.95	3.84
*dmc* × Chinese Spring	2023	396	27	15:1	0.0013	3.84
	2024	368	20	15:1	0.795	3.84
*dmc* × Guomai 301	2023	380	21	15:1	0.702	3.84
	2024	585	50	15:1	2.858	3.84

^1^ When *df* is 1, the significance value of *P* *_0.05_ is 3.84.

**Table 3:** Gene annotation of differentially expressed genes in candidate intervals on 5B and 7A.

#	Gene	Annotation	Expression in *dmc*
1	TraesCS5B02G058800	Auxin-responsive protein IAA31	up
2	TraesCS5B02G059300	CBL-interacting protein kinase 4	up
3	TraesCS5B02G059800	Tuliposide A-converting enzyme 2, chloroplastic	up
4	TraesCS5B02G060200	Dolabradiene monooxygenase	up
5	TraesCS5B02G060300	Dolabradiene monooxygenase	up
6	TraesCS5B02G061600	11-beta-hydroxysteroid dehydrogenase A	up
7	TraesCS5B02G062500	Methylcrotonoyl-CoA carboxylase subunit alpha	up
8	TraesCS7A02G184200	Protein containing a Bric-a-Brac/Tramtrack/Broad (BTB) complex and an NPH3 domain (BTBN), regulation of auxin transport	down
9	TraesCS7A02G184600	Shikimate kinase domain-containing protein	up
10	TraesCS7A02G187800	Aquaporin NIP III subfamily protein	down
11	TraesCS7A02G188100	Amino acid transporter	down
12	TraesCS7A02G188400	Plant regulator RWP-RK domain-containing protein	down
13	TraesCS7A02G191400	Small GTP-binding protein OsRac3	down
14	TraesCS7A02G191700	Domain of unknown function DUF23	down
15	TraesCS7A02G196100	TGF-beta receptor, type I/II extracellular region family protein	down
16	TraesCS7A02G197900	Similar to CEL5=CELLULASE 5 (fragment)	down

## Data Availability

The original contributions presented in this study are included in the article. Further inquiries can be directed to the corresponding author.

## Supplementary Materials

The following supporting information can be downloaded at: https://www.mdpi.com/article/10.3390/genes15121652/s1, Table S1: Oligonucleotide probes used for FISH; Table S2: Primers used in the experiment and their sequence information.
